# Improving image quality on pediatric and neonatal radiography using AI-based compensation for image degradation

**DOI:** 10.1007/s11604-025-01775-9

**Published:** 2025-04-07

**Authors:** So Ode, Atsuko Fujikawa, Atsushi Hiroishi, Yuki Saito, Takao Tanuma, Daigo Suzuki, Yuichi Sasaki, Hidefumi Mimura

**Affiliations:** 1 Department of Radiology, St. Marianna University School of Medicine, 2-16-1 Sugao, Miyamae-Ku, Kawasaki, Kanagawa 216-8511 Japan; 2 Imaging Center, St. Marianna University School of Medicine, 2-16-1 Sugao, Miyamae-Ku, Kawasaki, Kanagawa 216-8511 Japan

**Keywords:** Radiography, Pediatric radiology, Artificial intelligence, Noise reduction

## Abstract

**Purpose:**

To evaluate the impact of an AI-based, noise reduction technique for compensation of image degradation on pediatric and neonatal chest and abdomen radiography using a visual grading analysis.

**Materials and methods:**

Forty-six consecutive cases of pediatric and neonatal chest X-rays were identified for the quality evaluation. The images underwent AI-based noise reduction processing (Intelligent NR, Canon Inc.). All the images were randomized, and were evaluated from 1 to 4 for image quality by three board-certified radiologists in consensus. A score of “1” indicated the desired anatomy or features were not seen, “2” indicated quality between one and three, “3” indicated adequate quality, and “4” indicated higher than required image quality. A Wilcoxon signed rank test was used to assess the significant difference between images from conventional noise reduction versus those from the AI-based noise reduction.

**Results:**

The images processed with the INR(Intelligent NR) noise reduction had a higher image quality than the conventionally processed images, with a significant difference between the two groups (*p* < 0.05).

**Conclusion:**

The AI-based noise reduction technique improved the image quality of pediatric and neonatal chest and abdominal radiography significantly.

## Introduction

Since its introduction in the 1990s, digital radiography (DR) has been one of the most common pediatric imaging modalities due to its improved image quality and lower radiation dose compared to conventional imaging methods [1]. It is commonly used in various evaluations of the lung field and abdomen, as well as central venous line, and tube placement. Therefore, improvements in radiographic quality can enhance these diagnostic capabilities. However, ionizing radiation doses should be based on the principle of “as low a dose as reasonably achievable” (ALARA) [2] and as such need to be low, particularly in newborns, where their high radiosensitivity increases the risk compared to adults from the detrimental effects of radiation [3]. Current medical X-ray images are susceptible to degradation from noise, which is caused by quantum mottle from a limited incident dose. In pediatric imaging, it is important to improve image quality by reducing noise without increasing the incident dose. Noise reduction processing is an important technique to solve this problem, and several previous studies have reported new image noise reduction techniques to reduce image quality degradation caused by decreased incident dose [4–7]. However, conventional noise reduction processing is based on rules set by humans to separate the signal from the noise in images, which limits processing performance and, depending on the imaging area and conditions, may cause loss of the original subject’s signal if priority is given to noise processing. In recent years, however, highly accurate noise reduction processing techniques have been developed through the application of artificial intelligence (AI) technology. A new noise reduction process based on AI technology (Intelligent NR, Canon Inc.) uses DeepLearning to repeat machine learning of noise reduction processing, enabling the construction of highly accurate noise reduction processing algorithms [8].

Although the application of AI technology to improve image quality in various modalities and post-processing grid-like software in radiography has been reported in the past [9, 10], there are no reports, to our knowledge, of its application to noise reduction processing in pediatric radiography. The purpose of this study is to determine whether Intelligent NR (INR) can produce diagnostic images of portable radiographs of neonates and pediatrics taken in a clinical situation, and whether it can improve image quality compared to conventional methods.

## Materials and method

### Imaging acquisition

**Fig. 1:**
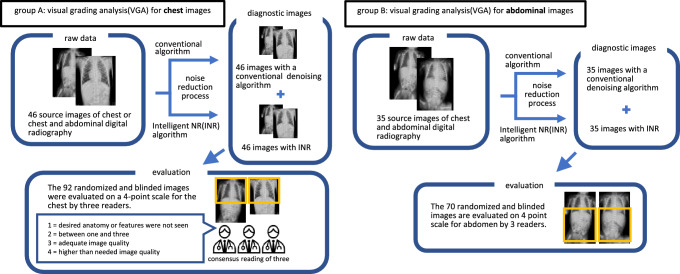
Study design for the evaluation of image quality for each group

Fifty images were randomly selected from 937 chest or chest and abdomen images in supine position taken with the portable digital radiography system (Sirius Starmobile tiara airy, HITACHI) in the National Intensive Care Unit (NICU) or general pediatric ward between June 1, 2021 and November 1, 2021. Of these, 4 images were excluded, including images in which the subject was not within the range or images for the purpose of confirming the location of various devices such as endotracheal tubes, nasogastric tubes, or intravascular lines, that were taken within a short period under the same conditions. Finally, a total of 46 images were retrospectively registered, including 11 chest and 35 chest and abdominal radiographs taken for 35 patients aged 0–50 months (22 males and 13 females). The entrance surface dose (ESD) was calculated for each image for the given values of tube voltage (kilovoltage peak:kVp) and tube current (milliampere-seconds:mAs) using the program software (Surface Dose Evaluation Code, Sdec). All 46 radiographs were registered in Group A for the chest evaluation and 35 chest and abdominal radiographs were registered in Group B for the abdominal evaluation. All images were processed using radiography software (CXDI Control Software NE, Canon Inc.) for noise reduction using INR and the conventional algorithms. One processed image was generated for each of the two noise reduction processes from a single raw image. Consequently, 92 images were generated from 46 raw images in Group A and 70 images from 35 raw images in Group B. These images were used for the image quality evaluation by radiologists after randomization and blinding (Fig. 1). Patient data and technical parameters of each group are shown in Table 1.

**Table 1:** Patient and technical examination data

	Group A	Group B
Number of patients, sex(male/female)	35 (22/13)	25 (16/9)
Age (month), median (min–max)	0.8 (0.0–50.1)	0.3 (0.0–32.8)
Number of images	46	35
Tube voltage (kVp), median (min–max)	52 (50–70)	50 (50–70)
Tube current (mAs), median (min–max)	1.2 (0.5–3.2)	1.2 (1.0–2.0)
ESD (μGy), median (min–max)	48.8 (22.8–117.5)	48.8 (36.6–74.6)

*kVp kilovoltage peak, *mAs* milliampere-seconds, *ESD* entrance surface dose

### Image evaluation

The images were transmitted to a picture archiving and communication system (PACS) workstation (RapideyeCore, Canon Medical Systems) and read on a Liquid Crystal Display (LCD) monitor (RadiForce RX650, EIZO).

Each image was randomized and blinded. The overall image quality of each image was evaluated for the chest region in Group A and the abdominal region in Group B by a consensus of three radiologists with at least eight years of experience, using Visual Grading Analysis (VGA) [11] with a score from 1 to 4, (1 = desired anatomy or features were not seen, 2 = between one and three, 3 = adequate image quality, 4 = higher than required). The anatomical structures used as evaluation factors for each group were determined with reference to the guidelines [2] (Table 2).

**Table 2:** Evaluation factors in each group

Group A	Group B
Pulmonary vessel	Bowel gas and wall interface
Retrocardiac vessel	Diaphragm
Trachea and bronchi	Lateral abdominal walls
Diaphragm	Soft tissue interface
Soft tissue interface	Cortex and trabecular bone (vertebrae, ribs and pelvis)
Cortex and trabecular bone (vertebrae and ribs)	Graininess
Graininess (granular noise)	

The window width and level for the images was freely adjustable by the reading physician.

### Statistical analysis

The VGA scores obtained for each image were compared between the conventional noise reduction-processed images and those processed with AI noise reduction by a Wilcoxon’s signed rank test using the statistical analysis software (JMP^®^ Pro 15, SAS Institute Inc.) for Groups A and B.

## Results

**Fig. 2:**
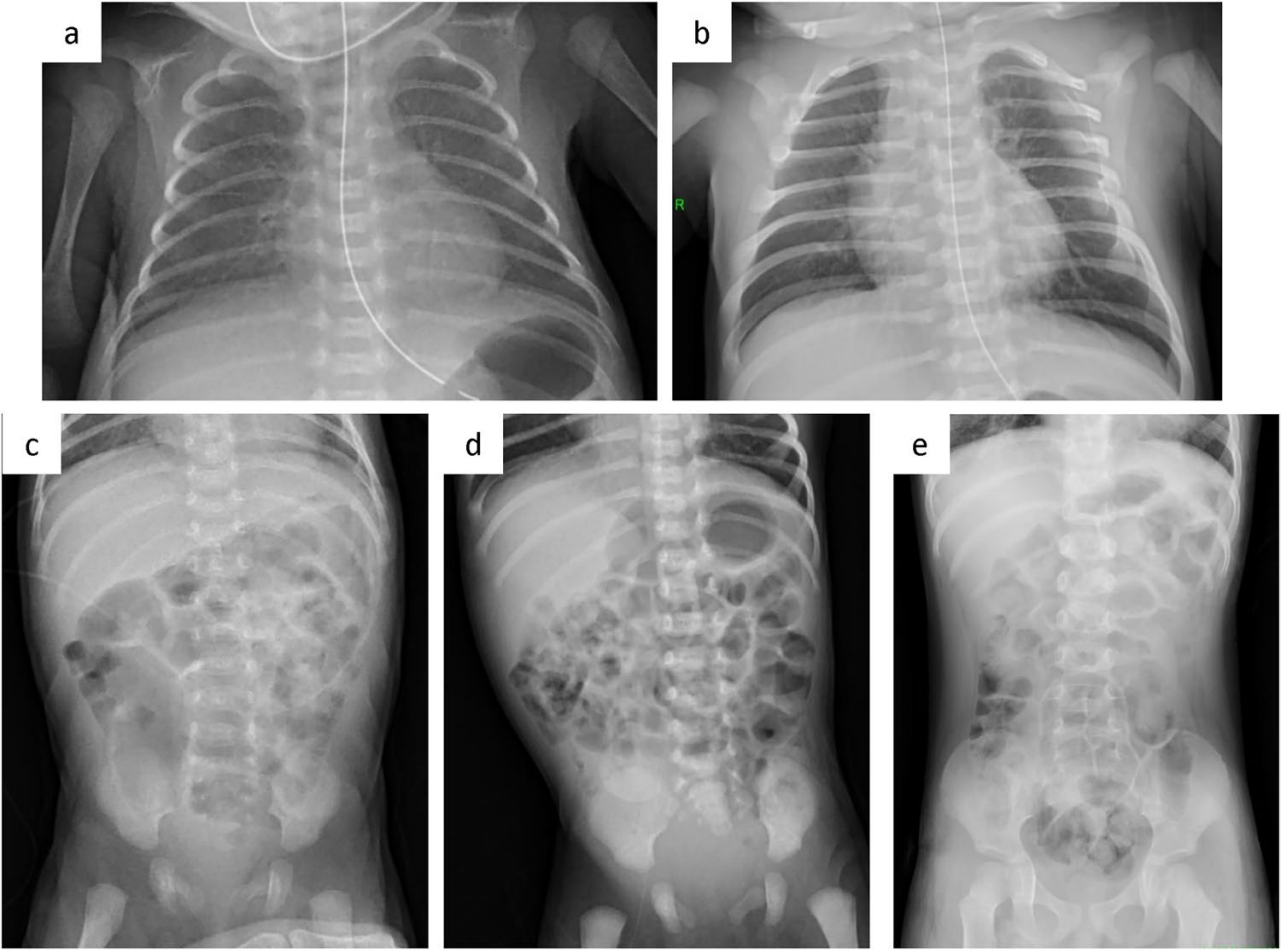
Examples of VGA scoring for processed images. Chest VGA score 3 (**a**). Processed by conventional algorithm. Granular noise is seen in the soft tissue and lung fields. Bone cortex and diaphragmatic margins are partially obscured. VGA score 4 (**b**). Processed by Intelligent NR(INR). Soft tissue, lung fields, bone cortex, and diaphragm are all clearly delineated. Abdominal VGA score 2 (**c**). Processed by conventional algorithm. Strong granular noise is seen in the soft tissues over a wide area, with obscured borders of the bone cortex and intestinal wall, and obscured bone beam structures. VGA score 3 (**d**). Processed by conventional algorithm. Granular noise is present, but only partially obscuring the bony structures and intestinal wall delineation. VGA score 4 (**e**). Processed by INR. Graininess is not prominent and soft tissues, bowel wall, bony structures, and diaphragm are clearly defined

**Fig. 3:**
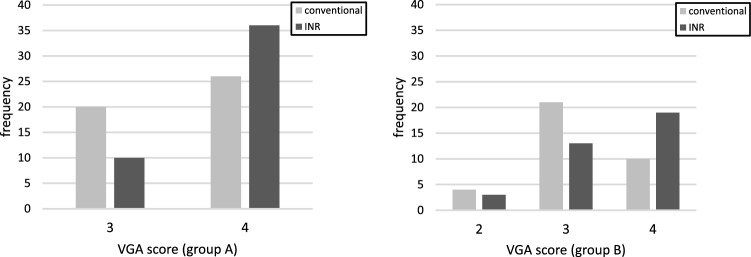
Frequency of VGA scores for each group

**Fig.4:**
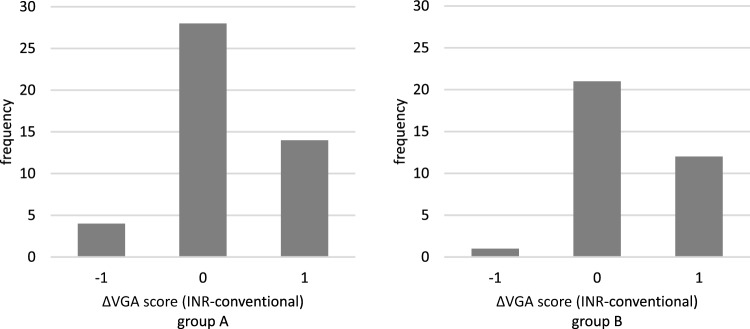
Difference in VGA scores between each noise reduction algorithm

Table 1 shows the patient age range and examination parameters. Figure 2 shows examples of VGA scoring for images with noise reduction processing. Figure 3 shows the results of VGA scoring after processed by noise reduction algorithm for each group. Figure 4 shows the distribution of the difference in scores due to differences in noise reduction processes for the same image for each group. The VGA score in Group A ranged from 3 to 4, while in Group B, the VGA score ranged from 2 to 4 (Fig. 3). The mean difference in VGA scores for the INR-processed images over the conventional noise reduction images in Group A (plain chest radiographs) was 0.217 (95% CI 0.041–0.394). The mean difference in Group B was 0.286 (95% CI 0.089–0.482). A Wilcoxon signed rank test showed that the scores from the INR images were significantly higher (*p* < 0.05) for both groups than those processed by conventional noise reduction (Table 3).

**Table 3:** Average VGA scores after noise reduction for each group

Group	VGA (mean ± SD)		Difference of mean score (95% CI)	*p*
	INR	Conventional		
A	3.78 ± 0.42	3.57 ± 0.50	0.217 (0.041–0.394)	0.0167*
B	3.46 ± 0.66	3.17 ± 0.62	0.286 (0.089–0.482)	0.0057*

^*^p < 0.05, the significance of the differences was tested with a Wilcoxon’s signed rank test

**Fig. 5:**
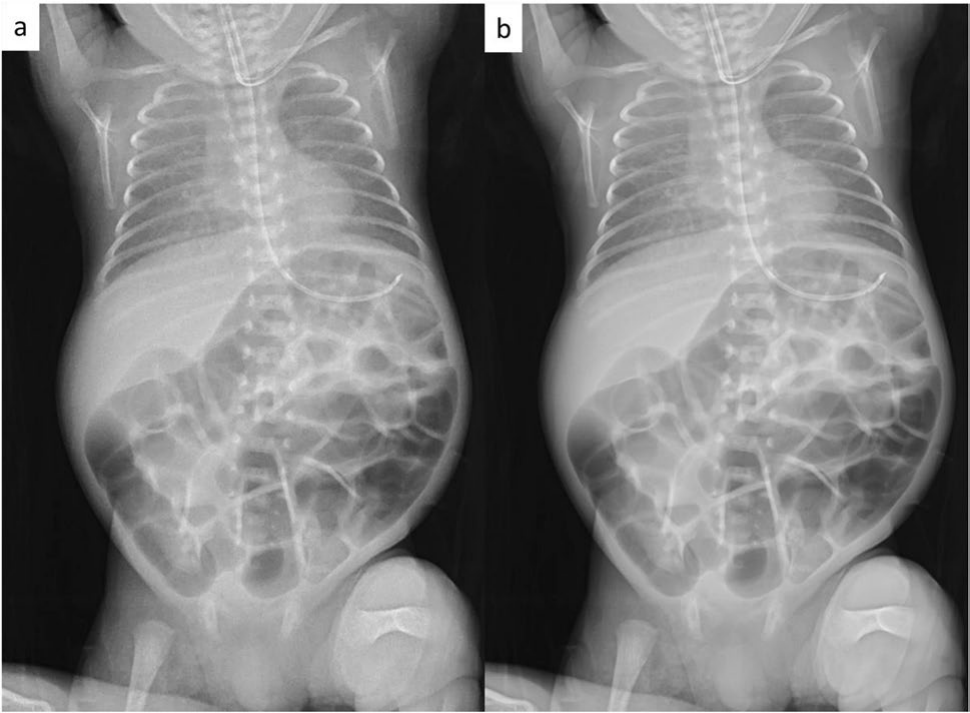
The differences in the image quality between each process on the same image. Compared to conventional noise reduction (**a**), INR (**b**) shows improvement in granular noise in the chest and abdomen, and clarification of lung fields, soft tissue, intestinal wall, bone cortex and trabecular structures

**Fig. 6:**
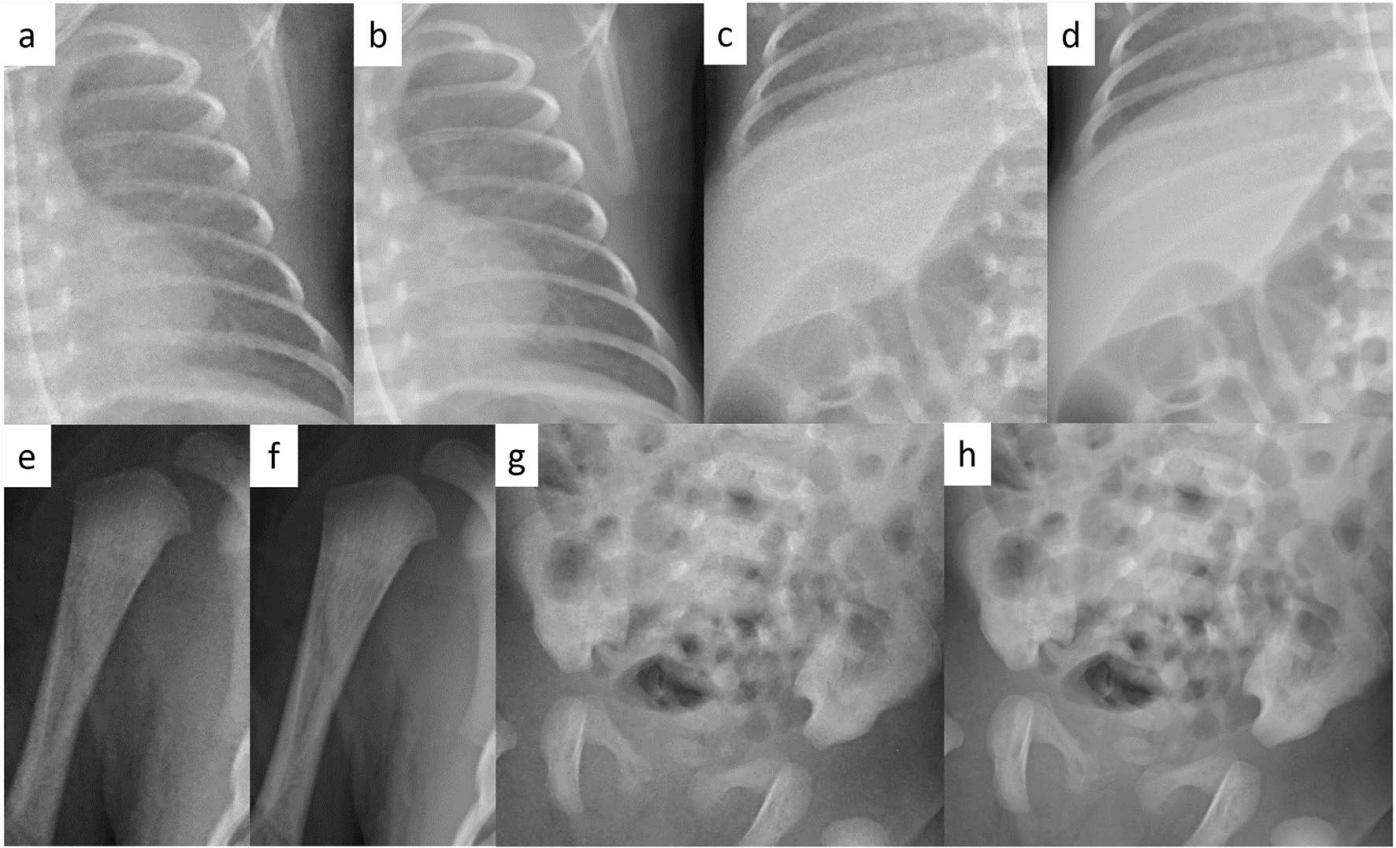
The differences in image quality between each process in each areas of the same image. In contrast to the conventional process (**a**, **c**, **e**, **g**), INR (**b**, **d**, **f**, **h**) shows a clearer lung pattern, a marked reduction in the mottled noise signal in the upper abdomen, and clearer bone cortex and trabecular structures

Examples of images obtained with conventional noise reduction versus INR are shown in Figs. 5 and 6.

## Discussion

The main noise in digital radiography is caused by quantum mottle. Noise reduction is post-processing of the captured image to improve the effect of this noise and improve image quality. Conventional noise reduction processes have improved the accuracy of processing by modifying rules created by humans, but they have the problem of losing the original signal of the subject. The INR with AI technique used in this study is based on a mathematical model of the complex noise reduction process, obtaining the following process. (1) images are randomly selected from a large database of clinical images prepared in advance. (2) Add noise and create a set of training data as input. The image before adding noise is used as the correct image. (3) Compare the inference result of noise elimination by the neural network and the correct answer, and calculate the loss. (4) Update the parameters of the neural network to reduce the loss. By repeating the above processes (1) to (4), it is possible to learn the noise reduction process by DeepLearning and build a mathematical model that enables separation of the signal and noise of a subject more complex and more accurately than rules created by humans [8].

In this study, for portable radiographs of neonates and pediatrics of 0–50 months, INR was shown to be capable of producing images of diagnostic quality and to significantly improve image quality compared to conventional noise-reduction processing. To the best of our knowledge, this is the first report of AI-based noise reduction applied to portable radiography of neonates and pediatrics.

Another report on the improvement of radiographic image quality using AI techniques have reported that the performance of post-processing techniques is unlikely to differ for children weighing less than 10 kg, such as newborns, when scatter noise is removed by software [10], but in this study, a significant improvement in image quality was observed despite the fact that most of the subjects were newborns. The reasons for this are considered to be that the effect of noise due to scattered radiation is smaller in children than in adults, that noise due to quantum mottle cannot be reduced only by removing scattered noise using software, and that the number of cases in this study was small.

In this study, while there was a tendency for image quality to improve with INR compared to conventional noise reduction algorithms, in both the chest and abdominal groups, it was found that image quality decreased in some radiographs processed with INR compared to conventional algorithm (Fig. 4). Possible causes include the fact that, although INR improves graininess to some extent, it may not sufficiently improve image quality in other evaluation items related to image quality assessment, the possibility that image quality deteriorates due to factors different from conventional algorithms in the case of INR using machine learning, and the possibility that there is variation in VGA scores based on visual assessment. If we want to more objectively examine the impact of differences in noise reduction processing on image quality assessment factors, we could consider evaluating the score assessment factors separately, but this is something we would like to consider in the future.

There are several limitations to this study. First, the number of cases was rather small. Second, the effects of age and body size were not evaluated. Third, differences in image quality assessment between radiologists and between sites were not evaluated.

In conclusion, this study suggests that a new noise reduction method based on AI technology can improve the image quality of chest or abdominal digital radiography in neonates and pediatrics as compared to conventional noise reduction methods.
